# Teratogenic Effects of Diatom Metabolites on Sea Urchin *Paracentrotus lividus* Embryos

**DOI:** 10.3390/md8040950

**Published:** 2010-03-30

**Authors:** Giovanna Romano, Antonio Miralto, Adrianna Ianora

**Affiliations:** Stazione Zoologica Anton Dohrn, Villa Comunale 80121 Napoli, Italy; E-Mails: antonio.miralto@szn.it (A.M.); adrianna.ianora@szn.it (A.I.)

**Keywords:** diatom, oxylipins, sea urchin, fatty acids, development arrest, apoptosis, TUNEL, teratogens

## Abstract

The diatom-derived polyunsaturated aldehydes (PUAs), 2*-trans*,4-*trans*-decadienal, 2-*trans*,4-*trans*-octadienal, 2-*trans*,4-*trans*,7-octatrienal, 2-*trans*,4-*trans*-heptadienal, as well as tridecanal were tested on early and later larval development in the sea urchin *Paracentrotus lividus*. We also tested the effect of some of the more abundant diatom polyunsaturated fatty acids (PUFAs) on development, in particular 5,8,11,14,17-eicosapentaenoic acid (EPA), one of the main precursors of diatom PUAs, as well as 4,7,10,13,16,19-docosahexaenoic acid (DHA), 6,9,12,15-octadecatetraenoic acid (stearidonic acid), 6,9,12-octadecatrienoic acid (γ-linolenic acid) and 9,12-octadecadienoic acid (linoleic acid). PUAs blocked sea urchin cell cleavage in a dose dependent manner and with increasing chain length from C7 to C10 PUAs, with arrest occurring at 27.27 μM with heptadienal, 16.13 μM with octadienal, 11.47 μM with octatrienal and 5.26 μM with decadienal. Of the PUFAs tested, only EPA and stearidonic acid blocked cleavage, but at much higher concentrations compared to PUAs (331 μM for EPA and 181 μM for stearidonic acid). Sub-lethal concentrations of decadienal (1.32–5.26 μM) delayed development of embryos and larvae which showed various degrees of malformations depending on the concentrations tested. Sub-lethal concentrations also increased the proportion of TUNEL-positive cells indicating imminent death in embryos and larvae. Using decadienal as a model PUA, we show that this aldehyde can be detected spectrophotometrically for up to 14 days in f/2 medium.

## 1. Introduction

**Figure 1:**
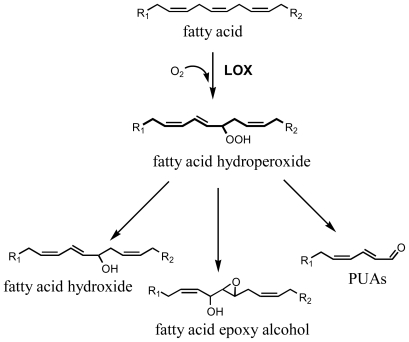
Biosynthetic sketch for the synthesis of oxylipins from fatty acids *via* lipoxygenase enzymes (LOX) in marine diatoms. R_1_ represent methyl terminal part and R_2_ the carboxylic end of C16 or C20 fatty acid precursors. R_1_ and R_2_ may differ in terms of length and degree of unsaturation depending on the position of O_2_ addition by LOX in the fatty acid molecule (modified from Fontana *et al*. [6]).

Diatoms are the dominant photosynthetic organisms in the world’s oceans, accounting for up to 40% of annual productivity at sea [1] and 25% of global carbon-fixation [2]. These microscopic, unicellular algae have traditionally been considered as a good food for plankton primary consumers, consisting mainly of small crustacean copepods that dominate the zooplankton, and in the transfer of carbon to higher trophic levels including important fisheries. However, their beneficial role in supporting marine food chains has been challenged with the discovery that some diatom species produce polyunsaturated aldehydes (PUAs) having cytotoxic and antitumoral activity ([3] onwards). PUAs are the end-products of a lipoxygenase/hydroperoxide lyase metabolic pathway [4] initiated by damage to algal cells, as occurs through grazing by predators. Cell damage activates lipase enzymes, which liberate polyunsaturated fatty acids (PUFAs) from cell membranes that are immediately oxidized and cleaved within seconds to form PUAs and a plethora of other metabolites collectively termed oxylipins [5,6] (Figure 1). The specific type and quantity of oxylipins produced differs between species and strains due to a variety of precursor PUFAs and enzymes [4–7], with variable effects on zooplankton grazers [8].

Oxyplins, and PUAs in particular, have important biological and biochemical properties including the disruption of gametogenesis, gamete functionality, fertilization, embryonic mitosis, and larval fitness and competence [9]. Although the effects of such toxins are less catastrophic than those inducing poisoning and death of predators, they are none-the-less insidious occurring through abortions, birth defects and reduced larval survivorship. Such antiproliferative compounds may discourage herbivory by sabotaging future generations of grazers, thereby allowing diatom blooms to persist when grazing pressure would otherwise have caused them to crash.

Diatom blooms are a potential energy source for benthic organisms as well, but the sinking of diatoms to the sediment at the end of the bloom may have negative consequences on reproductive activity in residing populations. Previous studies on benthic invertebrates have shown that PUAs inhibit fertilization processes, reduce larval fitness and induce teratogenesis in several broadcast-spawning species (see [9] for a review). For example, sea urchin gametes incubated in the diatom PUA decadienal showed impaired fertilization success due to inhibition of both sperm motility [10,11] and pronuclear fusion [12]. Arrest of cell cleavage has been reported by various authors in both *Paracentrotus lividus* and *Sphaerechinus granularis* eggs treated with decadienal [3,13,14]. At concentrations higher than the dose required to arrest cell cleavage progression, decadienal was found to induce apoptotic events in *P. lividus* embryos as demonstrated by means of a suite of techniques including the evaluation of caspase-3-like protease activity [15]. In addition, Hansen and co-workers [12] have demonstrated that decadienal is able to inhibit tubulin polymerization, DNA synthesis and cyclin B/Cdk1 kinase activity, leading to the arrest of the cell cycle in *S. granularis* early embryos.

Here we compare the effects of different diatom-derived PUAs, namely *2-trans*,4-*trans*-decadienal, 2-*trans*,4-*trans*-octadienal, 2-*trans*,4-*trans*,7-octatrienal, 2-*trans*,4-*trans*-heptadienal, and the saturated aldehyde tridecanal [16,17] on early and later larval development in the sea urchin *Paracentrotus lividus*. Apoptosis induction in the pluteus stage after chronic exposure to decadienal is evaluated for the first time by means of TUNEL staining. Since it has been suggested that free PUFAs may negatively affect reproductive success in sea urchins [18], we also tested the effect of some of the more abundant diatom PUFAs on sea urchin development, in particular 5,8,11,14,17,-eicosapentaenoic acid (EPA), one of the main precursors of diatom oxylipins, as well as 4,7,10,13,16,19-docosahexaenoic acid (DHA), 6,9,12,15-octadecatetraenoic acid (stearidonic acid), 6,9,12-octadecatrienoic acid (γ-linolenic acid) and 9,12-octadecadienoic acid (linoleic acid). The deleterious teratogenic effects of PUAs on sea urchin embryos are compared with those on other marine invertebrates.

## 2. Results and Discussion

### 2.1. Effect of PUAs on Sea Urchin Cleavage and Hatching

**Figure 2:**
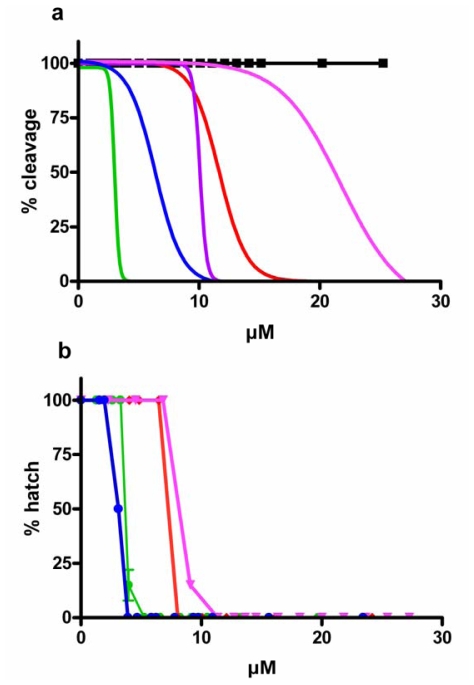
(a) Cleavage inhibition in sea urchin embryos following PUAs treatment. (b) Percentage of hatched sea urchin larvae. Sea urchin embryos were treated at increasing concentrations of 2-*trans*,4-*trans*-decadienal (
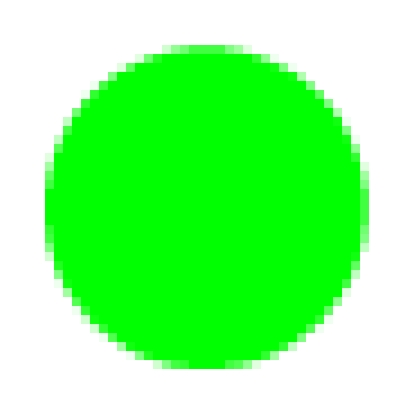
), aldehyde mix (see text) (
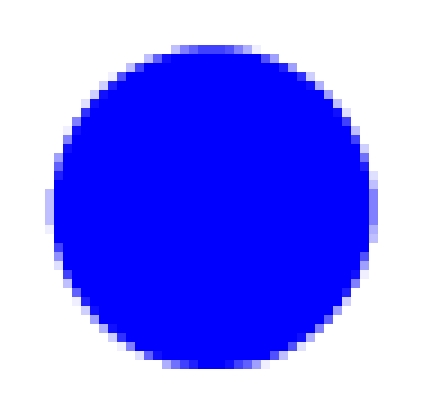
), 2-*trans*,4-*trans*-octadienal (
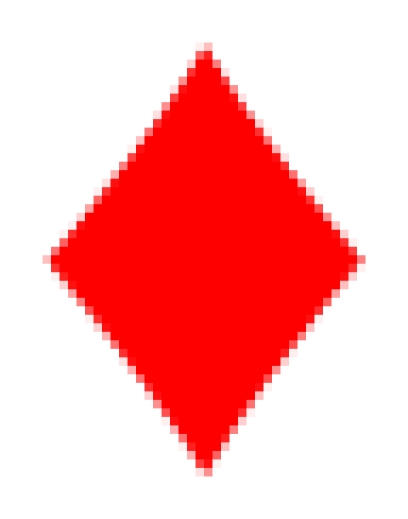
), 2-*trans,*4-*trans*,7-octatrienal (
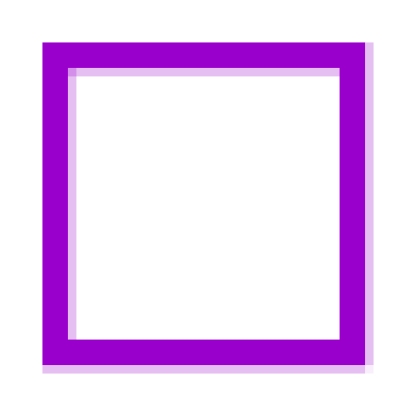
), 2-*trans*,4-*trans*-heptadienal (
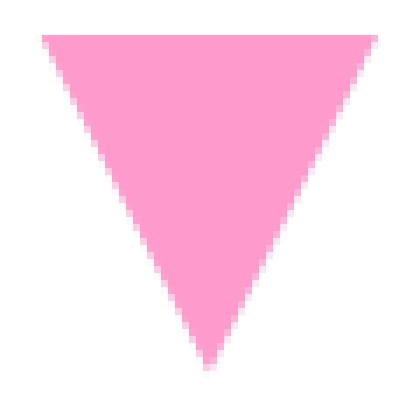
) and tridecanal (▪). Values (means ± S.D.; N = 600) are the results of three different experiments.

Sea urchin eggs have been extensively used to rapidly screen for bioactive compounds interfering with cell division processes [19] since they can be obtained in large numbers, may be fertilized under controlled conditions, and have synchronous development. Here we confirm the deleterious effect of PUAs such as 2-*trans*,4-*trans*-decadienal (decadienal), 2-*trans*,4-*trans*–octadienal (octadienal), 2-*trans*,4-*trans*,7-octatrienal (octatrienal), 2-*trans*,4-*trans*-heptadienal (heptadienal) on early and later developmental stages of *P. lividus.* PUAs blocked sea urchin cell cleavage in a dose dependent manner, but at different concentrations depending on the chain length of the molecules. Figure 2a reports the percentage of *P. lividus* embryos that were blocked after incubation with test compounds added to seawater at concentrations ranging from 0.658 to 32 μM. Percentage blockage of cell cleavage increased with increasing chain length from C7 to C10 PUAs, with arrest occurring at 27.27 μM with heptadienal, 16.13 μM with octadienal, 11.47 μM with octatrienal (which was slightly more active compared to octadienal), and 5.26 μM of decadienal. The saturated aldehyde tridecanal, also found in diatoms, did not interfere with first cleavage up to 25 μM. Higher concentrations were not tested due to solubility problems for this compound in sea water.

Previous studies conducted on *Sphaerechinus granularis* embryos [13,14] showed the same trend in activity-structural relationships. Adolph and coworkers [13] reported that only aldehydes bearing an α,β- or α,β,γ,δ-unsaturated structural element were active, whereas saturated and unsaturated aldehydes, which lack such a Michael acceptor system, exhibited no activity at all. The same authors did find an inactive α,β,γ,δ-unsaturated aldehyde, 9-oxo-nona-5*Z*,7*E*-dienoic acid, which, presumably due to intermolecular interactions, was not inhibitory. They found that the minimum dose of decadienal to inhibit 100% of embryo cleavage was about 20 μM, higher than the value reported in the present work. Pohnert [14] calculated an IC_50_ value for decadienal of 7.3 μM, similar to the value of 3.01 μM (95% CI = 2.96 to 3.06 μM) we have obtained in our experiments. Hansen [12] found that decadienal blocked cell divisions in newly fertilized eggs of the sea urchin, *S. granularis*, in a dose-dependent manner with an IC_50_ value of 1.3 μM in experiments with low egg density. This difference in IC_50_ values reported by different authors for the same species, as in the case of *S. granularis*, might be explained by variations in egg densities used in the experiments which determine the extent of interactions between PUAs and nucleophilic sites on both the egg surface or in the cytosol [11]. For the present study we constantly used an egg concentration of circa 120 eggs mL^−1^ in 5 mL wells as described in the material and methods section. The activity of decadienal on *P. lividus* embryos, expressed as IC_50_, is also comparable to that obtained by Hansen for *S. granularis*, although IC_50_ calculated for the latter species was somewhat lower. In test studies conducted in our laboratory, we found that *S. granularis* embryos appear to be more sensitive to the action of aldehydes (data not shown) compared to *P. lividus* using the same experimental conditions.

We also tested the effect of PUAs on sea urchin hatching success and found that all three PUAs exerted a very strong dose-dependent effect, with decadienal showing somewhat stronger effects than the other two aldehydes (Figure 2b). At concentrations of circa 3.0 μM, decadienal reduced hatching viability to <50%, but with octadienal and heptadienal, hatching viability was >90%. Total inhibition of hatching viability occurred at concentrations of 3.95 μM decadienal, 8.08 μM octadienal and 11.36 μM heptadienal, confirming that longer-chained aldehydes had somewhat stronger effects on hatching viability than shorter-chained aldehydes. It is interesting to note that no synergic effect on cleavage inhibition and only a slightly increased effect on egg hatching viability occurred when decadienal, octadienal and heptadienal were added as a mixture to the medium containing a 1:1:1 ratio of the three molecules.

When tested at concentrations below the concentration required to block cleavage in 100% of embryos, PUAs retarded development and induced malformations (see below). Embryos incubated in decadienal at concentrations above or equal to 3.95 μM were unable to develop beyond the blastula stage.

### 2.2. Effect of PUFAs on Embryo Cleavage and Hatching

When diatoms are damaged, the first enzymes to be activated are lipases that liberate a massive amount of free PUFAs. Among these, some PUFAs that are precursors for PUA production *via* lipoxygenase activity may be potentially harmful to developing embryos and larvae. Sellem and co-workers [18] described abnormal development in embryos and larvae of the sea urchin *Paracentrotus lividus* incubated with the fatty acid octadecapentaenoic acid (C18:5 n-3) derived from a dinoflagellate belonging to the genus *Gymnodinium*. Since these authors found a delay in development rate during the cleavage stage, we decided to test the effect of some commercially available PUFAs, namely 5,8,11,14,17-eicosapentaenoic acid (EPA C20:5 n-3), one of the main precursors of diatom oxylipins, as well as two other omega 3 fatty acids present in considerable quantities in diatoms, 4,7,10,13,16,19-docosahexaenoic acid, (C22:6 n-3, DHA), and 6,9,12,15-octadecatetraenoic acid (C18:4 n-3, stearidonic acid). We also tested 6,9,12-octadecatrienoic (C18:3 n-6, γ-linolenic acid) and octadecadienoic (C18:2 n-6, linoleic acid) acids, two omega 6 fatty acids that are very common in higher plants, but rare in diatoms.

**Figure 3:**
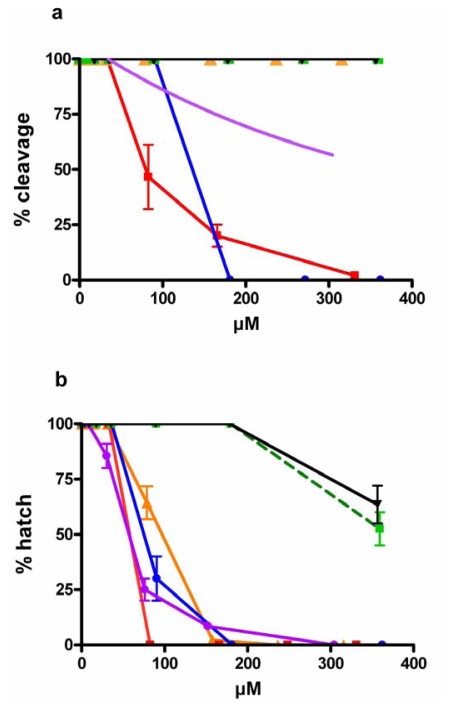
(a) Cleavage inhibition in sea urchin embryos following PUFAs treatment at increasing concentrations. (b) Percentage of hatched sea urchin larvae with increasing concentrations of fatty acids. EPA (
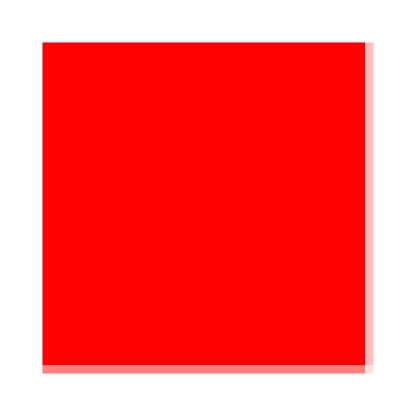
), EPA-ME (
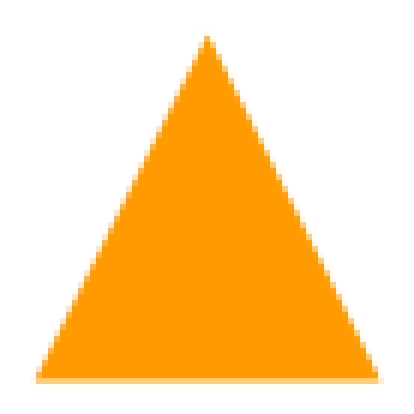
), DHA (
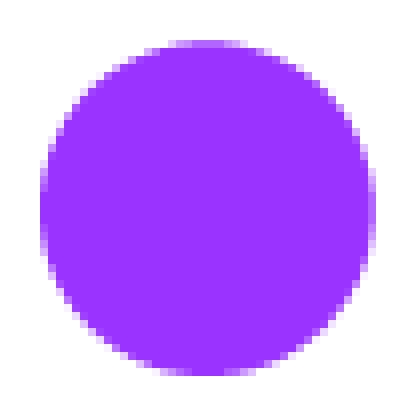
), stearidonic (
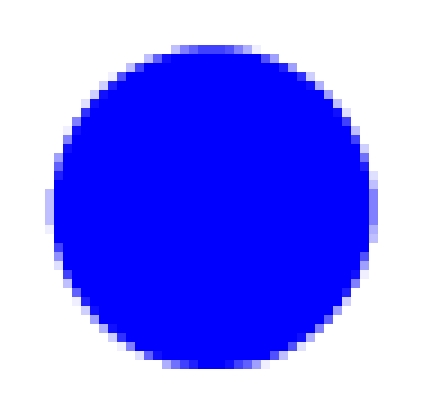
), linoleic (▾) and γ-linolenic (
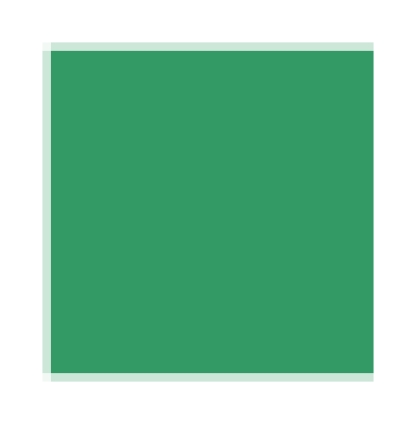
) acids. Values (means ± S.D.; N = 600) are the results of three different experiments.

Of the PUFAs tested, only EPA and stearidonic acid blocked cleavage, with this latter molecule showing the greatest activity (Figure 3a). Since cleavage was delayed depending on the concentrations tested (not shown), we recorded the percentage of first cleavage after 90 min, when almost 100% of control embryos had reached the 2-blastomere stage. After 90 minutes from fertilization, EPA completely blocked embryo cleavage at a concentration of 331 μM. In the case of DHA at 304 μM, only 34% of the embryos underwent first cleavage. The greatest activity was recorded with stearidonic acid that inhibited cleavage in 100% of embryos at 181 μM. Surprisingly no activity was recorded after incubation with linoleic and γ-linolenic acids, even at the highest concentrations tested of 357 and 359 μM, respectively.

In general, the biological activity of PUFAs is linked to the alteration that they induce in cell membrane structures, modifying membrane protein function, and influencing passive and active ionic transport as described by Gamberucci *et al*. [20] in relation to calcium conductance. Altering cell ion balance and calcium conductance can ultimately lead to cell death. It is interesting to note that the fatty acids having effects on sea urchin development are omega 3 fatty acids, as already reported for octadecapentaenoic acid by Sellem and coworkers [18] who found a decrease in the percentage of first cleavage with increasing concentrations of 18:5 n-3 and total blockage at concentrations of this fatty acid ≥1 mM. A possible explanation for the difference in activity between the two groups of PUFAs tested may be the more pronounced tendency of the first group to form hydroperoxides due to the greater degree of unsaturation. Hydroperoxides formed from EPA have been shown to be very active in blocking copepod early development compared to aldehydes and other oxylipins [6]. It is reasonable to assume that the same effect is also found in sea urchin embryos. In this case the activity we found is not ascribed directly to the fatty acids, but to their derivatives produced by spontaneous oxidative processes.

Our results also highlight differences in the activity between EPA and its methyl ester. In this case, the drastic reduction in the activity of the methyl ester may be due to different physico-chemical properties of the two molecules, leading to different mechanisms of interaction with the cell membrane.

The effect of free fatty acids on the transition to swimming blastula stage was also assessed (Figure 3b). The PUFAs EPA, DHA and stearidonic acid interfered with the transition to the larval stage. The most active PUFA was EPA with 0% of hatched larvae at concentrations of approximately 83 μM, whereas the least active were linoleic and γ-linolenic acids that reduced hatching to 60 and 50% of the embryos at 357 and 360 μM, respectively. Also, in this case omega 3 fatty acids showed a more pronounced activity with respect to omega 6 PUFAs. The EPA methyl-ester reduced the hatching of larva to 0% at a concentration of 158 μM, indicating that it was as active as PUFAs in inhibiting hatching. Other mechanisms may be responsible for this effect and additional studies are required to clarify these findings.

If we compare the effect of PUFAs on sea urchin development with that of PUAs, it is clear that aldehydes have a more pronounced and specific effect on embryonic and larval development, with at least one order of magnitude higher activity in blocking development compared to PUFAs.

### 2.3. Effect of PUAs on Paracentrotus lividus Larval Development

**Figure 4:**
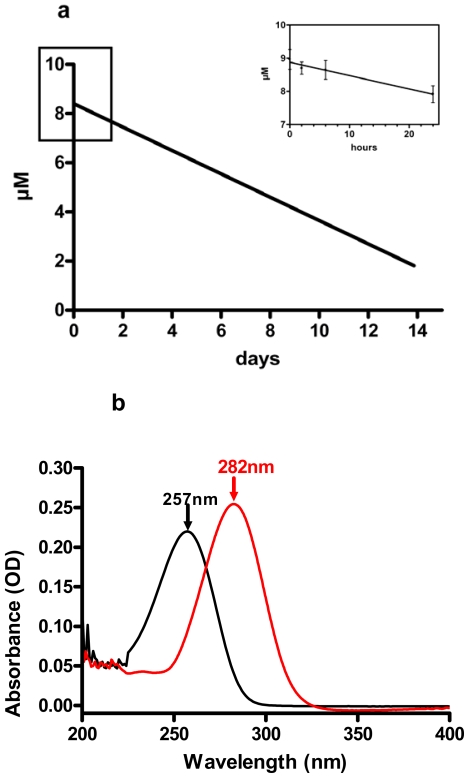
(a) Variation with time in decadienal concentration in f/2 medium. Each point is a mean value of three replicates calculated from a standard curve. (b) UV spectrum in f/2 medium of decadienal (red) and decadienoic acid (black).

In our new experiments using decadienal as a model PUA, we show that this aldehyde can be detected for several days in f/2 medium (Figure 4a), at least under the experimental conditions used for these measurements. Decadienal was incubated in natural 0.22 μm filtered sea water enriched with f/2 [21] nutrients at 8.96 μM starting concentration. The incubation was performed in a closed culture flask.

Variations in decadienal concentration were determined spectrophotometrically and followed with time. Figure 4a shows that initial concentrations of decadienal did not change significantly in f/2 for at least six hours, and only by 11.70% of initial concentrations after 24 hours (insert in Figure 4a), diminishing gradually thereafter from 7.92 (±0.3 SD) to 4.63 (±0.017) μM after seven days of incubation under natural light conditions, at 20 °C in a closed vessel. The UV spectrum of decadienal in f/2 showed a maximum absorbance value at 282 nm and its profile did not change with time even if absorbance decreased (not shown). This suggests that no appreciable conversion to the corresponding decadienoic acid occurs since this fatty acid has a UV spectrum with an absorbance maximum at 257 nm (Figure 4b). The enzymes involved in PUA synthesis have already been shown to remain active for 45 minutes after cell-wounding ([22], Figure 4 in [5]) resulting in high local concentrations of PUAs. Our new results indicate that this cloud of decadienal can remain relatively stable for days unless decadienal reacts with other organic molecules present in the environment. Volatilization could also potentially reduce the permanence of PUAs in solution, but this process is probably more important at the sea surface. The implications are that local concentrations of PUAs may be high enough to potentially impact fertilization success and embryonic fitness of marine organisms, including sea urchins.

Benthic eggs and larvae may come into contact with diatom PUAs in the field at the end of a bloom, with the mass sinking of diatoms to the sediment. Hence it is important to understand the fate of these compounds within the benthic realm. It has been suggested, for example, that benthic organisms respond to substances released by diatoms which act as cues to induce synchronous spawning [23], but not much is known regarding successive recruitment of benthic juveniles after the bloom. Environmental exposure to PUAs may be dietary or by direct encounter with spawned gametes, particularly if spawning coincides with diatom blooms. Senescent diatoms are known to undergo lysis and release lipoxygenase-end products [24] that can generate a microenvironment in which gamete fertilization occurs, and embryos and larvae develop and grow.

Due to the patchy nature of phytoplankton at sea, it is reasonable to expect high local concentrations in the proximity of breakage of diatom cells. Ribalet [25] estimated that such concentrations were well within the significant range for affecting growth and performance of surrounding organisms. Ultimately, to solve such questions, in situ measurements of aldehydes are needed. This is expected to represent a key step to understand the ecological role of these compounds in marine ecosystems.

**Figure 5:**
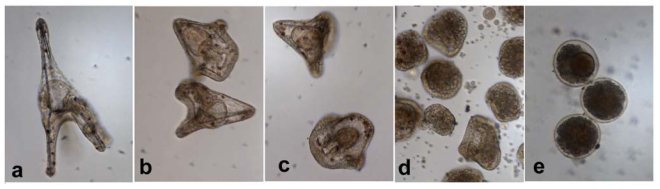
*Paracentrotus lividus* 48 hours plutei after incubation in decadienal at 1.32 (b), 2.63 (c), 3.95 (d) and 5.26 μM (e) compared to control embryo (a).

Incubation of newly fertilized eggs in concentrations of decadienal lower (1.32–5.26 μM) than those inducing cell blockage (6.58 μM) increased the number of abnormal sea urchin plutei and delayed the development of larvae or embryos which showed various degrees of malformations depending on the concentrations tested (Figure 5). At lower concentrations, malformations (Figure 5b,c) were less severe with a shortening of the apical spicules and arms. However at higher concentrations, larvae were similar to blastula and gastrula stages, showing severe abnormalities (Figure 5d) or blebbing associated with apoptosis (Figure 5e).

**Figure 6:**
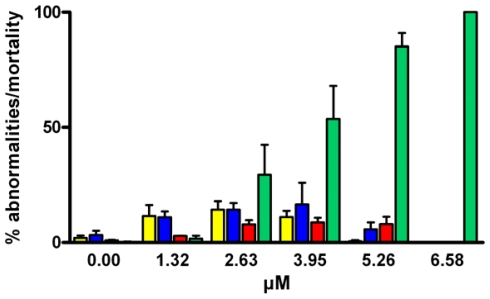
Percentage of abnormal sea urchin larvae following 48 hours treatment with decadienal ranging from 1.32–6.58 μM. Control is reported as 0 μM decadienal concentration. Yellow bars = retarded larvae; blue bars = abnormal pluteus larvae; red bar = abnormal gastrulae and blastulae; green = dead pre-hatched embryos. Values (means ± S.D.; N = 600) are the results of three different experiments.

The percentage of abnormal larvae after 48 hours of treatment with decadienal is reported in Figure 6. Decadienal at 1.32 μM induced an increase in the number of retarded (11.64% ± 8.2 SD) and abnormal plutei (11.08% ± 4.4 SD) compared to controls. At 2.63 μM decadienal, larvae were morphologically very similar to the gastrula stage and a considerable number of dead and pre-hatching embryos appeared (up to 29.4%), with a great variability among replicates (±22.42 SD). The number of dead embryos increased to 53.7% (±24.7 SD) at 3.95 μM of decadienal and 85.1% (±24.7 SD) of embryos were not able to survive at 5.26 μM.

Studies on the effect of PUAs on benthic organisms such as tunicates [26], echinoderms and polychaetaes [11,27] indicate that their effect could be as severe as in planktonic animals such as copepods. In the tunicate *Ciona intestinalis*, sub-lethal concentrations of 0.2 μg mL^−1^ (1.32 μM) decadienal induced the formation of abnormal tadpole larvae which showed developmental aberrations ranging from effects on sensory organ pigmentation, reduction in elongation of the tail, and blockage before the gastrula stage. Sea urchins also begin to show malformations at these concentrations. Caldwell and co-workers reported morphological measurements of *Psammechinus miliaris* echinopluteus larvae following sub-lethal exposure to decadienal [27]. They found significant differences from controls after exposure to 0.1, 0.5 and 1 μg mL^−1^ decadienal concentrations (respectively 0.66, 3.29 and 6.58 μM), that are in the same range of decadienal concentrations reported to be active on *P. lividus* pluteus larvae. The same authors reported that polychaetae larvae are very sensitive to aldehydes [9]. They found that decadienal induced morphological abnormalities in 9 day old larvae of *Nereis virens* exposed to this aldehyde during embryogenesis at concentrations of 0.01 and 0.05 μg mL^−1^ (0.066 and 0.329 μM respectively). These values are lower than those required to induce abnormalities in sea urchins. These findings indicate differences in sensitivity to aldehyde exposure among benthic organisms. On the other hand, in pelagic copepods the concentration to induce malformations and apoptosis seems to be somewhat higher, 1 μg mL^−1^ (6.58 μM) [28].

### 2.4. TUNEL Assay

**Figure 7:**
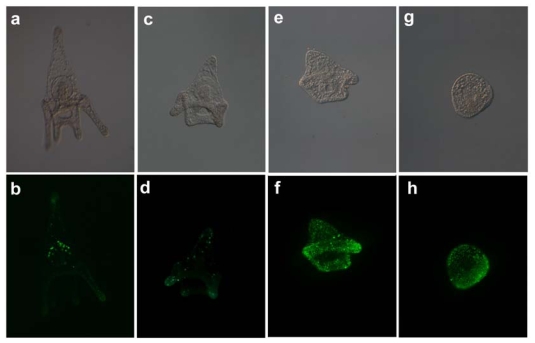
Tunel positive embryos incubated for 48 hours in decadienal at increasing concentrations (lower row) and corresponding images at transmitted light (upper row). Control Pluteus (a,b). Larvae after 48 hours incubation in decadienal 1.32 (c,d), 2.63 (e,f), 3.95 (g,h) μM.

Apoptosis has been shown to play two major roles during development, removing damaged cells during embryogenesis and sculpturing tissues during morphogenesis and metamorphosis [29]. But apoptosis is also induced in cells exposed to various stress factors including toxicants, pollutants and heat shock. Several authors have in fact reported both physiological and induced apoptosis in sea urchin embryos (reviewed by Agnello and Roccheri [30]). Previous studies conducted in our laboratory demonstrated that the PUA decadienal was able to induce apoptosis in early *P. lividus* embryos at concentrations five times higher than the dose required to induce 100% cleavage inhibition [15]. Apoptosis has been revealed using different techniques both in sea urchin and copepod embryos. In the present work we report results obtained on the induction of apoptosis at sub-lethal concentrations of decadienal after chronic exposure. Plutei exposed to decadienal were stained with the TUNEL fluorescent kit, specific for the detection of apoptosis. Plutei incubated in 1.32 μM decadienal showed somatic regions that were positively labeled, indicating occurrence of apoptosis in those tissues (Figure 7c,d). The morphological aspect of the larvae compared to controls (Figure 7a,b) suggest a reduction in developmental rate and spicules elongation since the oral arms were shorter and the apical region was reduced. Control plutei were also apoptotic but in different areas of the body compared to treated embryos. Thus, the effect of decadienal at 1.32 μM was to induce a delay in development in treated embryos. At higher concentrations, it also increased the proportion of TUNEL-positive cells in a dose dependent manner. At 2.63 μM approximately 80% of larvae appeared to be positive in the entire body, presenting a more pronounced delay in the development of the arms (Figure 7e,f). At 3.95 μM, embryos were quite similar to a late blastula and were apoptotic-positive throughout the entire body (Figure 7g,h).

**Figure 8:**
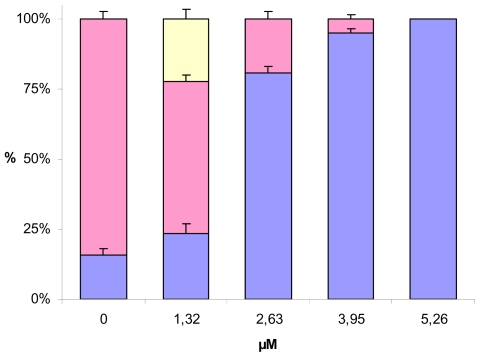
Percentage of TUNEL-positive embryos treated with increasing concentrations of decadienal. The percentage of larvae negative to TUNEL staining is shown in yellow. Pink indicates the percentage of embryos with nuclei that were positive to TUNEL localized in the arms and intestine. Blue indicates the percentage of larvae with apoptotic nuclei in the entire body. Values are means of three different experiments for approximately 200 embryos for each data point.

An increase in the number of apoptotic cells in the body following treatment with toxins and heavy metals has been observed in various invertebrate embryos [30]. It has also been shown that embryos slow down or suspend development and eliminate affected cells that might compromise the developmental program. In our studies decadienal not only slowed down development but also induced apoptosis in a dose dependent manner. This is shown in Figure 8 where we calculated the proportion of larvae presenting apoptotic regions in the entire body. At 1.32 μM decadienal, the number of embryos presenting a high degree of apoptosis was more or less the same as in the control. It is interesting to note that at this concentration larvae that were completely negative to TUNEL staining were also recorded suggesting the possible alteration of the cell cycle and apoptotic machinery. Already at 2.63 μM decadienal, the proportion of embryos presenting apoptotic nuclei in the whole body increased considerably reaching almost 80% of the total number of embryos examined. At 3.95 μM decadienal, there was a stronger effect and almost all of the larvae were positively stained.

Data available in the literature indicate that diatoms producing volatile compounds have a range of possible effects on benthic organisms. In particular the PUA 2-*trans*,4-*trans*,7-*cis*-decatrienal has been shown to act as a repellent for freshwater crustacean grazers [31]. Similar compounds are suspected to play a role in locating habitats for aquatic insects [32] and nematodes [33]. Several studies indicate that benthic invertebrates can detect and differentiate volatile oxylipins released from diatoms [31,34] and utilize them as food-finding cues. These studies suggest that diatom PUAs act as infochemicals, shaping the complex network of relationships between microalgae and their grazers. Our results indicate that even low concentrations of PUAs affect the developmental program in sea urchin embryos, with evident malformations and apoptosis induction suggesting that most of these embryos are destined to die. Following diatom bloom termination the concentration of PUAs can be high enough to compromise the recruitment of future generations of these organisms. What remains poorly understood is if sea urchins actually feed on diatoms at the end of the bloom thereby compromising their fitness.

## 3. Experimental Section

### 3.1. Aldehydes and Fatty Acids Stock Solutions

The polyunsaturated aldehydes (PUAs) 2-*trans*,4-*trans*-heptadienal, 2-*trans*,4-*trans*-octadienal, 2-*trans*,4-*trans*-decadienal and tridecanal were obtained from Sigma-Aldrich Inc. (Milano, Italy). Stock solutions of PUAs were prepared by diluting in absolute methanol (J.T. Baker, Deventer, Holland). The effective PUA concentration in stock solution was assessed spectrophotometrically (HP spectrophotometer model 8453) by using the molar absorption coefficient of 31,400 at 274 nm wavelength [35]. Work solutions were obtained by diluting appropriate volumes of stock solutions in sea water (SW) and mixing carefully to ensure optimal dispersion. Methanol had no effect on sea urchin cleavage up to 10% methanol in sea water. The amount of aldehyde solution in each test was kept equal to or below this threshold. Synthetic octatrienal were kindly provided by G. d’Ippolito and stock solutions were prepared as described above for other aldehydes.

The polyunsaturated fatty acids (PUFAs) eicosapentaenoic acid (EPA C20:5, n-3) and its methyl ester (EPA-ME), docosaexaenoic acid (DHA, C22:6, n-3), stearidonic acid (C18:4 n-3), linoleic (C18:2, n-6) and γ-linolenic acid (C18:3, n-6) were obtained from Sigma–Aldrich Inc. (Milano, Italy) as ethanol solutions. Stock solutions of PUFAs were prepared by diluting the concentrate substances in absolute methanol. Work solutions were prepared as described above.

### 3.2. Decadienal Stability in Nutrient Enriched Sea Water

Decadienal stock solution was prepared by diluting a known amount of the substance (determined by weight) in methanol. The methanolic solution was further diluted in autoclaved f/2 medium [21] to achieve decadienal concentration ranging from 0.66 to 32.89 μM. A standard curve was generated by reporting the absorbance at 274 nm of decadienal as a function of its concentration (each determination was performed in triplicate).

To verify decadienal stability, solutions were prepared in triplicate at a starting concentration of 8.96 μM in 250 mL f/2 and incubated in a polystyrene culture flask closed with a screw cap. The UV spectrum and absorbance reading at 274 nm were assessed with time. Decadienal concentration was determined on the basis of a standard curve.

Decadienoic acid was obtained by oxidation of commercial decadienal. The aldehyde (60 mg) was dissolved in ethanol (1.5 mL) in the presence of an acqueous solution of silver nitrate (189 mg in 0.5 mL of water); the reaction mixture was stirred for three hours at room temperature, filtered and extracted between hydrochloric acid (5 M) and diethyl ether; the organic phase was evaporated and the product was obtained (64 mg) [36]. Decadienoic acid solutions and UV spectrum were obtained as described for decadienal. Absorbance determinations were performed using a HP model 8453 spectrophotometer.

### 3.3. Sea Urchin Gamete Collection

*Paracentrotus lividus* (Lamarck) sea urchins were collected during the breeding season by SCUBA diving in the Gulf of Naples and transported in an insulated box to the laboratory, within 1 h after collection, and maintained in captivity in well-aerated water at 18 °C in an open circuit. Living organisms were injected with 0.2 mL of 0.2 M acetylcholine (SIGMA-Aldrich, Milan, Italy) through the peribuccal membrane to induce gamete ejection. Spawned eggs were allowed to settle and were washed three times with 0.22 micron filtered sea water (FSW); eggs were then diluted to a final concentration of 3000 eggs mL^−1^. Concentrated sperm was collected ‘dry’, mixing samples from at least three different males and stocking undiluted at +4 °C. Sperm mix was diluted 10 μL in 10 mL SW just before insemination and an aliquot of circa 100 μL of this solution was added to 100 mL of egg suspension. At 2–4 min after sperm addition the eggs were checked for successful fertilization and excess sperm were removed by washing the eggs once with FSW.

### 3.4. Embryotoxicity Test

PUAs and PUFAs were tested on sea urchin early development. Soon after fertilization envelope elevation, approximately 500 embryos were transferred to tissue culture wells containing 0.658–32 μM) aldehydes final concentration in 5 mL FSW. All concentrations were tested on egg groups collected from three different females. Groups of eggs from the same animals were used as controls. To test the effect of the solvent, other groups of embryos were incubated in 10% methanol, which was the highest concentration of solvent used for incubation experiments. Embryos were kept at 20 °C in a controlled temperature chamber under 12:12 light:dark cycle.

Observations were made after circa 90 minutes from fertilization, when almost 100% of embryos were at the two blastomere stage. Cleavage inhibition was assessed by counting at least 200 embryos for each well. After 24 hours, the percentage of hatched larvae, as well as larval mortality was determined by counting at least 200 embryos for each well.

PUAs were also tested for their teratogenic effect after long term incubations. After 48 hours of incubation with test compounds, the number of abnormalities and mortality were assessed according to Pagano *et al.* [37]. Embryos at various stages were observed under a light microscope (Zeiss Axiovert 135TV). Pictures were taken using a Zeiss Axiocam connected directly to the microscope.

Statistical analysis and IC_50_ calculation were made by using the Prism software (GraphPad software inc. La Jolla, CA, USA).

### 3.5. Tunel Fluorescence Labeling (TUNEL)

*P. lividus* embryos were incubated as described above in decadienal at concentrations ranging from 1.32 to 6.58 μM for 48 hours. Pluteus larvae were fixed in paraformaldehyde 4% (SIGMA-Aldrich, Milan, Italy) in sea water for 2 hours at room temperature. Fixed sea urchin embryos were washed several times in phosphate saline buffer 10 mM at pH 7.4 (PBS) to remove paraformaldehyde and then incubated for 1 h in a solution of 0.1% Triton X-100 and 0.1% sodium citrate, at 4 °C. Apoptosis was assessed using TdT-mediated dUTP nick end labeling (TUNEL) (Roche Diagnostics) according to manufacturer’s instructions. After washing in PBS containing 1% bovine albumin serum (BSA, Sigma-Aldrich), treated samples were incubated at 37 °C for 90 min in TUNEL solution in a humidified chamber, in the dark. Control embryos were fixed and stained as described above. To obtain TUNEL-positive control samples, embryos were incubated for 10 min in 50 mM Tris-HCl, pH 7.5, 10 mM MgCl_2_, 0.1% dithiothreitol, containing 250 μg mL^−1^ DNase I (grade II from bovine pancreas; Boehringer GmbH, Mannheim, Germany) at room temperature. Negative controls were obtained by incubating embryos in label solution only, as recommended by the manufacturer. Whole-mount sea urchin embryos were observed with a Zeiss AxioImager M1 epifluorescence microscope. Pictures were taken using a Zeiss Axiocam connected directly to the microscope.

## 4. Conclusions

Our data show that diatom-derived aldehydes can compromise embryonic and larval development of benthic organisms such as sea urchins even at low doses. The most deleterious of the PUAs tested were the longer chain aldehydes such decadienal, confirming previous studies by other authors. We show that decadienal can be detected for up to 14 days in seawater at concentrations that induce cell damage to sea urchin embryos. Interestingly, not only aldehydes but common fatty acids in diatoms, such as stearidonic acid (C18:4 n-3) and eicosapentaenoic acid (EPA C20:5, n-3) also arrest cleavage, but at much higher concentrations. Another important aspect that has emerged from this study is the delay in development that PUAs exert on embryos at low concentrations. This may have serious consequences on survival and on the timing of many life processes including recruitment and mating. Until now most studies on prey-predator interactions in benthic organisms have focused on feeding deterrents with little attention to what happens when predators actually feed on their prey. Our results show that if they do, or if newly spawned eggs are exposed to these metabolites, recruitment may be compromised with possible effects on cohort size of the next generation.
